# Control of innate immunity and lipid biosynthesis in neurodegeneration

**DOI:** 10.3389/fnmol.2024.1402055

**Published:** 2024-07-25

**Authors:** Daniel R. Scoles, Stefan M. Pulst

**Affiliations:** Department of Neurology, University of Utah, Salt Lake City, UT, United States

**Keywords:** innate immunity, cholesterol biosynthesis, fatty acid biosynthesis, neurodegenerative disease, INSIG1, SREBP, STING, TBK1

## Abstract

The cGAS-STING innate immunity pathway and the SREBP-activated cholesterol and fatty acid synthesis pathway are abnormally co-regulated in neurodegenerative disease. Activation of STING signaling occurs at the endoplasmic reticulum (ER) membrane with STING anchored by INSIG1 along with SREBP and the sterol-bound SREBP cleavage activating protein (SCAP) when sterols are in abundance. When sterols are low, the INSIG-dependent STING pathway is inactivated and the SREBP-SCAP complex is translocated to the Golgi where SREBP is cleaved and translocated to the nucleus to transactivate genes for cholesterol and fatty acid synthesis. Thus, there is inverse activation of STING vs. SREBP: when innate immunity is active, pathways for cholesterol and fatty acid synthesis are suppressed, and vice versa. The STING pathway is stimulated by foreign viral cytoplasmic nucleic acids interacting with the cyclic GMP–AMP synthase (cGAS) DNA sensor or RIG-I and MDA5 dsRNA sensors, but with neurodegeneration innate immunity is also activated by self-DNAs and double-stranded RNAs that accumulate with neuronal death. Downstream, activated STING recruits TBK1 and stimulates the transactivation of interferon stimulated genes and the autophagy pathway, which are both protective. However, chronic activation of innate immunity contributes to microglia activation, neuroinflammation and autophagy failure leading to neurodegeneration. STING is also a proton channel that when activated stimulates proton exit from STING vesicles leading to cell death. Here we review the salient features of the innate immunity and cholesterol and fatty acid synthesis pathways, observations of abnormal STING and SREBP signaling in neurodegenerative disease, and relevant therapeutic approaches.

## 1 Introduction

Biochemical processes that protect cells from disease and infection are also processes that can become chronically activated in neurodegenerative disease. As such, these processes are prime targets in the development of therapeutics for neurodegenerative disease, and so, deep understanding of the relevant biochemical pathways is critical to therapeutic development. This review focuses on two interconnected pathways with disease-relevance, the STING pathway activated by viral nucleic acids upon stimulating nucleic acid sensors, and cholesterol and fatty acid synthesis that is activated by SREBP. These seemingly two unrelated pathways are connected at a regulatory hub that involves the protein INSIG1 and other regulatory proteins that remarkably operate to suppress cholesterol and fatty acid synthesis when the STING pathway is activated, and conversely suppress the STING pathway when the cholesterol and fatty acid synthesis pathway is activated. With neurodegenerative disease, neuronal death leads to the release of nucleic acids that can chronically activate the STING pathway and have negative consequences in cholesterol and fatty acid synthesis as well. Recently, a proton channel function was discovered for STING on Golgi-derived STING vesicles that when hyperactivated leads to cell death (Liu et al., 2023; Xun et al., 2024). Here we will review the functions of INSIG1 and SCAP in the regulatory hub that determine SREBP activity and synthesis of cholesterol and fatty acids vs STING activity and signaling in the innate immunity pathway that also signals to TBK1, a protein that is mutated in amyotrophic lateral sclerosis (ALS) (Freischmidt et al., 2017). There are several studies that describe the role of INSIG in regulating innate immunity, and INSIG and SREBP on regulating cholesterol and fatty acid signaling, but few that highlight the key proteins in a molecular decision-point that determines how these two signaling pathways are inversely controlled involving INSIG and SREBP in neurodegenerative disease. We describe these pathways and summarize our study of spinocerebellar ataxia type 2 (SCA2) mouse cerebellar transcriptomes as an example of relevance to ataxia and ALS.

## The PAMPs and DAMPs of innate immunity

The innate immunity system is a network of pathways with multiple functions affording protection to cells and organisms against pathogens. At the highest level the innate immunity system is stimulated by the activation of multiple pattern recognition receptors (PRRs) that recognize pathogen-associated molecular patterns (PAMPs), consisting of peptidoglycans, flagellin, lipid polysaccharides, double-stranded (ds) DNAs or dsRNAs from foreign viral or bacterial pathogens. Stimulation of PRRs ultimately has multiple outcomes including activation of the compliment system that enhances recognition and clearance of pathogens, activation of autophagy aiding in the recycling of cellular debris, stimulation of interferon (INF) expression, and production of proinflammatory cytokines. Innate immunity system PRRs can also be stimulated in the absence of pathogens by damage-associated molecular patterns (DAMPs), where the origins of DAMPs are host cells, and may include various cellular microparticles, nuclear DNA, mitochondrial DNA and RNAs (West et al., 2015; Magna and Pisetsky, 2016). In chronic diseases DAMPs are persistent, leading to maladaptive chronic stimulation of PRRs and cell death. PAMP-mediated activation of innate immunity occurs in immune cells (dendritic cells, B cells, and T cells), but in neurodegenerative diseases activation of the innate immunity pathway by DAMPs can also occur in microglia contributing to neuroinflammation and neuronal death, and PRRs are also expressed in astrocytes, neurons and endothelial cells of the brain and spinal cord (Kigerl et al., 2014).

**FIGURE 1:**
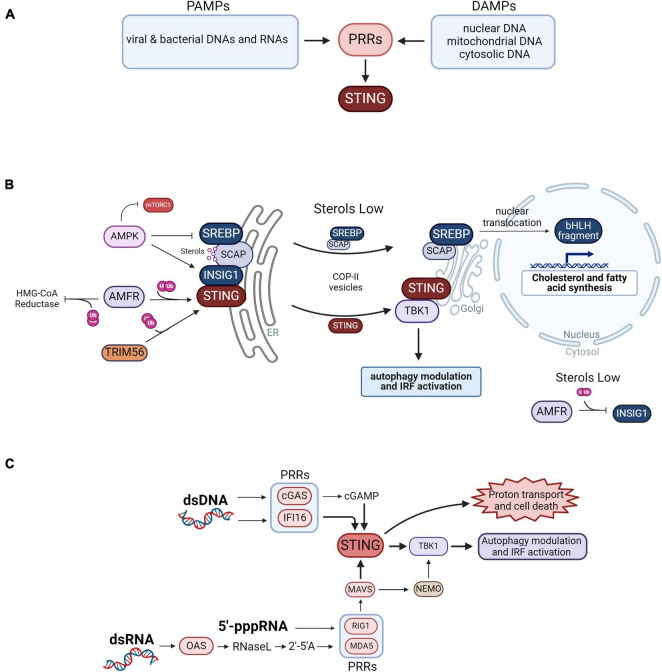
Control of innate immunity vs. lipid biosynthesis. **(A)** Summary of the PAMPs and DAMPs that activate pattern recognition receptors (PPRs) that signal to STING. **(B)** Coordinated activity of innate immunity vs. cholesterol and fatty acid synthesis is determined by sterol abundance and INSIG1 activity. STING dimers are anchored to the ER membrane by INSIG1 and by ubiquitination by AMFR. Ubiquitination of STING by TRIM56 also promotes its dimerization. When activated by DAMPS and PAMPS, STING translocates to the Golgi and recruits TBK1 to modulate autophagy by phosphorylating mTORC1, optineurin and p62. TBK1 also activates IRF signaling leading to the transactivation of type 1 interferons. AMFR also inhibits cholesterol synthesis by ubiquitinating HMG-CoA reductase leading to its proteasomal degradation. INSIG1 anchors SCAP bound to SREBP to the ER membrane when SCAP is bound to sterols, preventing cholesterol and fatty acid synthesis by blocking SCAP access to COPII vesicles. Phosphorylation of INSIG1 by AMPK prevents it from being ubiquitinated by AMFR, while phosphorylation of SREBP by AMPK inhibits its cleavage. When sterols are low in abundance the SREBP-SCAP complex is released from INSIG1 which is ubiquitinated by AMFR and degraded in the proteosome. The SREBP-SCAP complex passes to the Golgi apparatus via COPII vesicles where the SREBP bHLH domain fragment is cleaved away and translocated to the nucleus to transactivate genes for cholesterol and fatty acid biosynthesis. **(C)** Neurodegenerative disease-associated activation of STING by nucleic acid pattern recognition. In neurodegenerative disease, self- nuclear or mitochondrial DNAs interact with cGAS that then produces 2′3′cGAMP, a cyclic dinucleotide second messenger ligand recognized by STING. dsDNA can also stimulate IFI16 which then activates STING. The genomes of RNA viruses include 5′-triphosphorylated RNA termini (5′-ppp-RNA) that activate the RLRs RIG-I and MDA5. In neurodegenerative disease, self-dsRNA can also interact with OAS leading to the production of 2′-5′ oligoadenylate (2′-5′A), a second messenger that activates RNase L leading to the activation of RIG1 and MDA5. These RLRs then activate MAVS which then activates TBK1 in a STING-dependent manner. MAVS can also activate TBK1 in a STING-independent manner by way of NEMO. STING is also a proton channel and when hyperactive on STING vesicles leads to proton exit and cell death.

The principal innate immunity PRRs stimulated by PAMPs and DAMPs include the toll-like receptors (TLRs) and the NOD-like receptors (NLRs) which predominantly recognize cellular components of bacteria, C-type lectin receptors (CLECs), and the RIG-like receptors (RLRs) that are stimulated by viral RNAs and other nucleic acids. Stimulation of RLRs leads to activation of stimulator of interferon signaling (STING) that in turn activates the TANK binding kinase I (TBK1), resulting in interferon secretion in immune cells, and RLR activation has other consequences relevant to neurodegenerative disease. Figure 1A illustrates the PAMPs and DAMPs that stimulate PPRs activating the STING pathway.

## Coordinated STING vs. SREBP signaling

The metabolism of lipids and cholesterol are co-regulated by a mechanism linking STING and SREBP at the ER membrane. STING activation is coordinated with inactivation of SREBP regulated genes, and conversely, limiting the SREBP pathway results in increased STING signaling and activation of INFs. A more complete picture of this metabolic-inflammatory circuit was described in a 2015 study led by the laboratory of Steven Bensinger (York et al., 2015). Key findings of the study highlighting the inverse regulation of the STING vs. SREBP pathways included: (1) IFN treatment causes reduced SREBP pathway activation and increased cholesterol import; (2) Cells or mice null for the SREBP cleavage activating protein (*SCAP*) gene had elevated STING pathway activation; (3) Cells treated with a shRNA targeting *SREBP2* had increased abundance of transcripts in the innate immunity signaling pathway, determined by RNA-seq; and (4) Addition of exogenous cholesterol in cells null for *SCAP* attenuated type I INF signaling in a STING-dependent manner. The Bensinger group concluded that reduced flux in cholesterol synthesis constitutes a “danger” signal that triggers the type 1 INF response affording protection to the cell from viral infection. In the following paragraphs we describe how INSIG and SCAP function in a molecular decision point that determines activation of the STING pathway vs. the SREBP pathway.

## INSIG at the hub of the STING pathway and lipid biosynthesis

INSIG is a central player in activation of innate immunity vs. lipid synthesis (Figure 1B). The activation of lipid biosynthesis is associated with inhibition of the STING pathway and vice versa (York et al., 2015). Several factors determine the direction of the pathway, controlled by sterol abundance and INSIG status of activation that determines STING vs. SREBP activity.

### Sterols in abundance, INSIG1 activated

Signaling from activated RLRs leads to activation of STING which occurs in an INSIG-dependent manner. INSIG is also an SREBP retention factor that positions SREBP bound to SCAP at the ER membrane which inhibits lipid biosynthesis (Nohturfft et al., 2000; Yang et al., 2002; Sun et al., 2007). SREBP sequestration to this ER membrane-bound complex occurs when lipid synthesis is not needed, and is supported by SCAP binding to cholesterol which alters its conformation to favor its interaction with INSIG (Nohturfft et al., 2000; Yang et al., 2002; Adams et al., 2004; Sun et al., 2007).

### AMFR and AMPK, the reinforcers of STING dependent innate immunity vs. cholesterol and fatty acid synthesis

The E3 ubiquitin ligase AMFR/GP78 reinforces innate immunity signaling vs. cholesterol synthesis when sterols are in abundance. For STING activation it must be polyubiquitinated to position it to the ER membrane, which is accomplished by a complex of INSIG1 and AMFR (Wang et al., 2014). Simultaneously, the INSIG1-AMFR complex supports the polyubiquitination of HMG-CoA reductase, which catalyzes the rate-limiting step in cholesterol synthesis, leading to its degradation by the ubiquitin-proteasome system, preventing cholesterol synthesis (Song et al., 2005). Further reinforcement of innate immunity signaling vs. cholesterol and fatty acid synthesis is provided by AMP-activated protein kinase (AMPK). AMFR also ubiquitinates INSIG1 which can facilitate its rapid degradation when released from SCAP but otherwise is protected by SCAP interaction (Gong et al., 2006). Phosphorylation of INSIG1 by AMPK prevents AMFR interaction with INSIG1 and reinforces STING activation (Han et al., 2019). Simultaneously, AMPK phosphorylates SREBP inhibiting its cleavage and nuclear translocation ensuring inhibition of cholesterol and fatty acid synthesis (Li et al., 2011). Additionally, STING ubiquitination by the E3 ubiquitin ligase TRIM56 promotes STING dimerization, which is required for downstream activation of TBK1 (Tsuchida et al., 2010).

### Sterols in short supply, INSIG1 degraded

When sterols are low in abundance STING signaling is inhibited and lipid biosynthesis is activated. A low abundance of sterols causes the SREBP-SCAP complex to release INSIG1 which is then ubiquitinated by AMFR and rapidly degraded (Gong et al., 2006). Depletion of INSIG1 leads to inhibition of the STING-dependent innate immunity pathway (Wang et al., 2014). The SREBP-SCAP complex then translocates to the Golgi apparatus via COPII vesicles (Sun et al., 2007). Following this, SREBP is processed and its bHLH fragment translocates to the nucleus and transactivates genes containing the sterol regulatory element SRE leading to activation of cholesterol and fatty acid synthesis (Wang et al., 1994).

## The principal activators of STING

STING is a sentry protein in a critical nucleic acid-stimulated innate immunity pathway regulating the synthesis of type I interferons. There are multiple upstream activation points of the STING pathway, illustrated in Figure 1C. The principal pathway stimulated by dsDNA is known as the cGAS-STING pathway (Figure 1C). cGAS is stimulated by dsDNA to produce 2′3′cGAMP, a cyclic dinucleotide second messenger, that activates STING at the ER membrane (Sun et al., 2013; Wu et al., 2013). STING can also be activated by dsDNAs binding with IFI16, that then interacts with and activates STING (Unterholzner et al., 2010). Thereafter, activated STING interacts with TBK1 also at the ER membrane leading to its phosphorylation and signaling to its downstream targets. The origin of the dsDNAs can be viral or bacterial, but in neurodegenerative disease self-dsDNAs can lead to chronic activation of STING.

The STING pathway can also be stimulated by self-dsRNA and viral dsRNAs as illustrated in Figure 1C. In the RLR-STING pathway, the RLRs RIG-I and MDA5 are activated by 2′–5′ oligoadenylate synthase (OAS) family dsRNA sensors that process dsRNAs into 2′–5′ oligoadenylate (2′-5′A) that then activate RNase L which then activates RIG-I or MDA5 by a mechanism that has been characterized as poorly understood (Drappier and Michiels, 2015; de Reuver and Maelfait, 2023). RLRs can also be activated directly by single-stranded (ss) or dsRNAs bearing a 5′-triphosphate group (5′-pppRNA) that is typical of many viral RNAs (Lu et al., 2010; Abbas et al., 2013). Once activated, the RLRs then activate STING which then translocates to the Golgi and recruits TBK1 (Taguchi et al., 2021). This can occur in a mitochondrial antiviral-signaling protein (MAVS)-dependent manner (Weber et al., 2013) or MAVS can activate TBK1 in a STING-independent manner that is dependent on NF-kappa-B essential modulator NEMO (Figure 1C; Wang et al., 2012; Liu et al., 2013; Fang et al., 2017).

The recruitment of TBK1 to STING results in the phosphorylation of interferon regulatory factors (IRFs) by TBK1 and modulation of mTOR-dependent autophagy. Activated TBK1 phosphorylates IRF3 and IRF7 (Tanaka and Chen, 2012; Suschak et al., 2016). The IRFs then translocate to the nucleus to transactivate genes regulated by the interferon-sensitive response element (ISRE), which include genes encoding the type I interferons.

## STING signaling in neurodegeneration

Neuronal death in neurodegenerative disease leads to an accumulation of DAMPs including nuclear and mitochondrial DNAs and RNAs that chronically activate the RLRs and the STING pathway in both neurons and glia. There is reliable evidence for this in mice modeling Parkinson’s disease: *Pink1*^–/–^ or *Prkn*^–/–^ mice display a strong STING-dependent neuroinflammatory and innate immunity signature that can be alleviated by restoration of the abundance of either Pink1 or Parkin (Sliter et al., 2018). The loss of dopaminergic neurons in *Prkn*^–/–^ mice could also be normalized by reducing STING expression suggesting STING as a potentially effective therapeutic target for Parkinson’s disease (Sliter et al., 2018). Additionally, in ALS cytoplasmic aggregations of TDP-43 were seen to stimulate a rise of mitochondrial DNA in the cytoplasm activating cGAS and the STING pathway. This was observed in cellular and mouse models with mutant TDP-43 and confirmed in patient-derived induced pluripotent stem cell (iPSC) motor neurons and postmortem spinal cord tissues from ALS patients (Yu et al., 2020). Genes of innate immunity pathways regulated by STING are also activated in immune cells and cerebellar tissues from C9ORF72 ALS patients, but minimally when STING abundance was reduced (McCauley et al., 2020). The Niemann-Pick disease type C (NPC) protein NPC1 functions in trafficking STING to the lysosome where it is degraded. In *Npc1* knockout mice, STING signaling was upregulated and mice were characterized by Purkinje cell (PC) loss and ataxia motor phenotype, while genetic deletion of *Sting1* improved PC abundance and ataxia scores (Chu et al., 2021). Recently, a new proton channel function of STING was discovered on post-Golgi STING vesicles, and cell death results when this function is hyperactivated (Liu et al., 2023; Xun et al., 2024). Further research will be needed to determine if neuronal death results due to chronic activation of STING by DAMPs associated with neurodegenerative disease.

Abnormal fatty acid and cholesterol synthesis was also observed in human neurodegenerative diseases. Notably, NPC1 deficiency which, as described above, is associated with overactive STING, is also characterized by deficiency of cholesterol biosynthesis (Chu et al., 2021). Another example is Huntington’s disease (HD), that was characterized by reduction of cholesterol and fatty acid synthesis, associated with abnormal mutant HTT protein actions on the expression of SRE gene expression (Block et al., 2010). But it remains a complex problem, as elevated cholesterol biosynthesis was also observed in serum of ALS patients associated with neuronal death (Abdel-Khalik et al., 2017).

**FIGURE 2:**
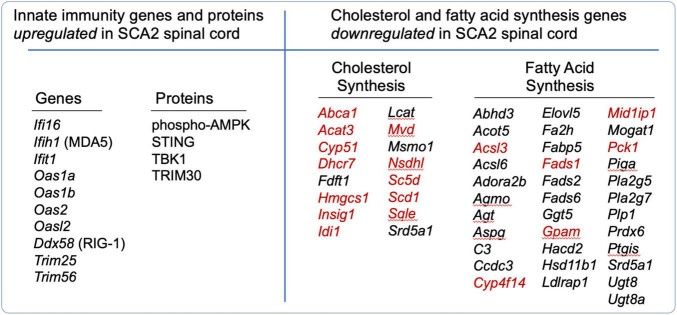
Differentially regulated genes (DEGs) and proteins that function in innate immunity and in cholesterol and fatty acid synthesis in SCA2 mouse spinal cord. DEGs with | log_2_(FC)| > 0.585, *P* < 0.05 are shown. Proteins were identified overabundant by western blotting. From Scoles et al. (2020). Red font indicates known SREBP targets, reviewed by Reed et al. (2008).

A prime example of the elegant complexity of innate immunity and cholesterol and fatty acid synthesis is SCA2. We found that innate immunity and cholesterol and fatty acid signaling were abnormally regulated in spinal cord (SC) transcriptomes of bacterial artificial chromosome (BAC) ATXN2-Q72 mice, or “SCA2 mice.” We performed transcriptome analysis of SCA2 mice using weighted gene co-expression network analysis (WGCNA) to identify relevant gene clusters (co-expression modules) with associated functional pathway genes (Scoles et al., 2020). We identified three significant gene expression modules. One was enriched in innate immunity pathway genes that were highly upregulated, while the other two modules included genes for cholesterol and fatty acid synthesis that were downregulated. Lists of selected differentially expressed genes in these pathways are provided in Figure 2, and complete lists can be found in the supplemental data of Scoles et al., 2020. Among the cholesterol and fatty acid biosynthesis genes, many were previously shown regulated by SREBP (Reed et al., 2008), suggesting they have the SRE promoter element. We have indicated these with red font in Figure 2. We also investigated SCA2 mice for key SC proteins by western blotting, revealing that STING was overabundant, TRIM30, a modulator of STING function was overabundant, AMPK was hyperphosphorylated, and TBK1 was overabundant (Figure 2). The results led to our deeper understanding on how these pathways are interconnected and their relevance to neurodegenerative disease.

Neurodegeneration leads to accumulation of DAMPs and chronic activation of STING and TBK1 and autophagy failure. Activated STING is well characterized for stimulating autophagy: STING can activate autophagy directly by lipidation of LC3 at STING vesicles in a non-canonical autophagy pathway (Gui et al., 2019; Liu et al., 2019; Fischer et al., 2020; Xun et al., 2024), or by activating TBK1 (Herhaus, 2021). TBK1 regulates autophagy by phosphorylating the autophagy receptors optineurin and p62 (Wild et al., 2011; Matsumoto et al., 2015; Prabakaran et al., 2018). Optineurin and p62 function in autophagic cargo selection and their dysregulation results in autophagy blockade and further accumulation of DAMPs, produced by among other things, breakdown of mitochondria concomitant with loss of mitophagy (Nakahira et al., 2011). TBK1 also modulates mTORC1, activating it by phosphorylation of S2159 on mTOR (Bodur et al., 2018) or inhibiting it by phosphorylation of S877 on Raptor (Antonia et al., 2019), and so chronic TBK1 activation disables autophagic and other metabolic pathways regulated by mTORC1 (Hasan et al., 2017). Mutations in TBK1 also lead to autophagy defects as well as neuroinflammation that underly ALS (Freischmidt et al., 2017; Oakes et al., 2017; Ye et al., 2019).

Our findings on SCA2 also support chronic autophagy as an underlying neurodegenerative disease defect. We had previously found that autophagy is abnormally inhibited in SCA2 patient fibroblasts and cerebellar and spinal cord tissues of SCA2 mouse models, as well as ALS and frontotemporal dementia (FTD) fibroblasts and mouse models associated with overabundance of the RNA binding protein STAU1 (Paul et al., 2018, 2021, 2023). STAU1 overabundance disables autophagy because it directly interacts the *MTOR* 5′-UTR to increase its translation (Paul et al., 2023). Autophagy failure in SCA2 aligns with overactive innate immunity seen in SCA2 mouse spinal cords suggesting chronic activation by accumulating DAMPs. Interestingly, STAU1 overexpression is also associated with increased abundance of the 5′-pppRNA-stimulated innate immune effector genes IFIT2 and IFIT3, and the dsRNA sensor OASL (Zhong et al., 2020), and STAU1 can enhance MDA5 and RIG1 binding to dsRNA (Ji et al., 2022).

A summary of neurodegenerative diseases with abnormal STING and cholesterol & fatty acid signaling is shown in Table 1.

**TABLE 1:** Neurodegenerative diseases with altered STING or cholesterol/fatty acid signaling.

Disease	Evidence	Citations
Amyotrophic lateral sclerosis (ALS)	STING pathway activation	McCauley et al., 2020; Yu et al., 2020; Marques et al., 2024
Frontotemporal dementia (FTD)	STING pathway activation	Marques et al., 2024
Parkinson’s disease (PD)	STING pathway activation	Sliter et al., 2018
Spinocerebellar ataxia type 2 (SCA2)	STING pathway activation, reduced cholesterol and fatty acid synthesis	Scoles et al., 2020
Huntington’s disease (HD)	Reduction of cholesterol and fatty acid synthesis	Block et al., 2010
Niemann-Pick disease type C (NPC)	STING pathway activation, impaired cholesterol biosynthesis	Chu et al., 2021

## Therapeutics targeting innate immunity and cholesterol and fatty acid synthesis

Decout et al. (2021) provides a comprehensive review of therapeutic compounds targeting the cGAS-STING pathway that potentially could be beneficial for neurodegenerative diseases characterized with overactive STING signaling (Decout et al., 2021). Metformin is an activator of AMPK and autophagy that has significant and beneficial effects on inhibiting the overactive cGAS-STING pathway (Chen et al., 2016; Ren et al., 2022). Metformin has been used in patients with fragile X syndrome (FXS), Alzheimer’s disease (AD), and Parkinson’s disease (PD) [reviewed in (Gantois et al., 2019)]. Of these patient studies, there was benefit for reducing risk of PD in a Taiwanese population (Wahlqvist et al., 2012). Metformin was also beneficial in various mouse models of neurodegenerative disease, including FXS, HD, AD, and PD [also reviewed in (Gantois et al., 2019)]. Importantly, metformin treatment of BAC-C9ORF72-500 transgenic mice was effective for improving molecular and immunohistological phenotypes in lumbar spinal cord, as well as open field and gait phenotypes (Zu et al., 2020). While we speculate that metformin reinforces the direct actions of AMPK on INSIG1 and SREBP as in Figure 1B, metformin is pleiotropic and also inhibits mitochondrial complex 1 and the Map/Erk pathway (Fontaine, 2018; Gantois et al., 2019).

Selective cGAS-STING inhibitors may also be beneficial for neurodegenerative disease, but have not been used systemically in humans. H-151 is a specific inhibitor of STING that might also be used for treating chronic innate immunity activation (Kobritz et al., 2022). Another specific inhibitor of STING is C-176, which inhibited microglial STING to normalize neuroinflammation activated by mitochondrial DNA release in an ischemic stroke mouse model (Kong et al., 2022). RU.521 is a specific cGAS inhibitor that likewise was effective for normalizing neuroinflammation in an ischemic stroke mouse model (Ding et al., 2022).

Upregulating SREBP might also be beneficial for diseases characterized by reduced cholesterol and fatty acid synthesis. LXR receptor agonists potentially have the ability to activate SREBP signaling (Zhang et al., 2012).

## Summary and conclusion

Control of innate immunity and the cholesterol and fatty acid synthesis pathways is maintained by INSIG1 which is positioned at the ER membrane by SCAP bound to SREBP when cholesterol is in abundance. When cholesterol is low in abundance INSIG1 is released and rapidly degraded while the SCAP-SREBP complex passes to the Golgi where SREBP is cleaved and translocated to the nucleus to activate SRE-regulated genes. In neurodegenerative disease abnormal autophagy is associated with inability to recycle cellular components leading to accumulating DAMPs and chronic activation of the cGAS-STING innate immunity pathway and STING-mediated LC3 lipidation. Therapeutics targeting STING and AMPK appear to be promising for treating neurodegenerative diseases with chronic innate immunity pathway activation.

## Author contributions

DS: Conceptualization, Data curation, Formal analysis, Funding acquisition, Investigation, Methodology, Project administration, Resources, Validation, Visualization, Writing−original draft, Writing−review and editing. SP: Conceptualization, Funding acquisition, Investigation, Methodology, Resources, Validation, Writing−review and editing.
